# Hypothetical Interventions on Cardiovascular Health Metrics for Abnormal Cognitive Aging: An Application of the Parametric g-formula in the CLHLS Cohort Study with 12 Years Follow-Up

**DOI:** 10.14283/jpad.2024.143

**Published:** 2024-07-09

**Authors:** S. Huang, Z. Zhao, S. Wang, Y. Xu, Z. Wang, J. Wang, H. Wang, X. Yu, Xiaozhen Lv

**Affiliations:** 1 Beijing Dementia Key Lab, Peking University Institute of Mental Health (Sixth Hospital), National Clinical Research Center for Mental Disorders, NHC Key Laboratory of Mental Health, Peking University, 51 Huayuan North Road, Haidian District, Beijing, 100191 China; 2 Department of Epidemiology and Biostatistics, School of Public Health, Peking University, 38 Xueyuan Road, Haidian District, Beijing, 100191 China; 3 Department of Pharmacy Administration and Clinical Pharmacy, School of Pharmaceutical Sciences, Peking University Health Science Center, Beijing, China

**Keywords:** Cognitive aging, cardiovascular health, g-formula, group-based trajectory model

## Abstract

**Background:**

Abnormal cognitive aging is closely related to dementia.

**Objectives:**

This study aimed to estimate the effect of cardiovascular health (CVH) metrics on abnormal cognitive aging.

**Design:**

A longitudinal cohort study.

**Setting:**

Participants were recruited from the Chinese Longitudinal Health Longevity Survey.

**Participants:**

A total of 3298 participants aged ≥65 years with normal cognitive performance at baseline were included.

**Measurements:**

Cognitive performance was measured by the Chinese version of the Mini-Mental State Examination (MMSE). CVH was assessed with six metrics, including hypertension, diabetes, exercise, body mass index (BMI), diet, and smoking. Group-based trajectory model was used to identify the trajectory groups of cognitive aging over 12 years (2002–2014 and 2005–2018). The parametric g-formula was applied to estimate the effect of each single six CVH metrics and their combinations on the 12-year cognitive aging trajectory.

**Results:**

Four trajectory groups of cognitive aging were identified: Stable-high (77.4%), Unstable (4.9%), Slow decline (11.1%), and Rapid decline (6.6%). Unstable, Slow decline, and Rapid decline trajectory groups were considered as abnormal cognitive aging (22.6%). Single interventions on hypertension, exercise, BMI, and diet could reduce the risk of abnormal cognitive aging. Moreover, the risk ratios of joint intervention on exercise, BMI, and diet for Unstable, Slow decline, and Rapid decline trajectory groups were 0.38 (95% CI: 0.30–0.48), 0.45 (95% CI: 0.37–0.54), and 0.3 (95% CI: 0.23–0.41), respectively.

**Conclusion:**

A considerable proportion of the participants experienced abnormal cognitive aging during their aging process. Interventions on these CVH metrics (i.e., exercise, BMI, and diet), which are fairly practical and feasible for older adults, may be effective strategies for preventing abnormal cognitive aging.

**Electronic Supplementary Material:**

Supplementary material is available in the online version of this article at 10.14283/jpad.2024.143.

## Introduction

Population aging has been a great challenge for health and social care worldwide due to declining fertility rates and increasing life expectancy (1). By 2050, it is estimated that approximately one-sixth of the global population will be aged 65 years and older (2). Since cognitive function is widely believed to be one of the most important capacities influencing the well-being of older adults, the maintenance of cognitive function is essential for healthy aging (3). Cognitive aging refers to longitudinal and dynamic cognitive changes throughout the aging process (4). A series of studies have shown that the trajectories of cognitive aging vary widely, and two to four trajectory groups of cognitive aging have been reported (5, 6). Most studies identified a relatively stable and high functioning trajectory group, and contrasted this were one or more groups that declined to varying extents or improved over time, which indicated significant interindividual heterogeneity in terms of cognitive aging (3). Notably, when abnormal, these cognitive changes can be considered symptoms of pathological conditions and are closely related to dementia (3). Thus, the need to identify the risk and protective factors of abnormal cognitive aging is becoming increasingly valued.

Cardiovascular health (CVH) metrics were first established by the American Heart Association (AHA) and include 7 modifiable risk factors (i.e., hypertension, diabetes, total cholesterol, exercise, body mass index, diet, and smoking) for cardiovascular diseases (7). According to a previous study (8), interventions involving overall CVH metrics may be more effective at reducing the risk of cardiovascular events than any single factor since these factors often coexist and share mutual causal pathways. Moreover, prior research also highlighted that CVH metrics had measurable health benefits beyond heart disease, and in particular altering cognitive aging (9). Recently, there also has been strong evidence that cardiovascular mechanisms are associated with the etiology of dementia, Alzheimer’s disease (AD) and vascular dementia (10). A meta-analytic study reported that the treatment of hypertension and hyperlipidemia could significantly reduce the incidence of dementia or AD (11). Research based on the Northern Manhattan Study demonstrated the benefits of ideal CVH promotion for cognitive aging (12). Several 1- to 2-year follow-up random clinical trials (RCTs) also showed a strong relationship between interventions on diet and exercise and cognitive decline (13, 14).

However, some studies investigating the effect of CVH metrics on cognitive aging showed contradictory results. In an Austrian RCT study, 24-month multidomain interventions on exercising, healthy diet, and antihypertension were found to have no beneficial effect on the prevention of cognitive decline (15). A study that harmonized data from 20 population-based cohorts across 15 countries (not including China) demonstrated significant ethno-regional differences in the associations of diabetes, total cholesterol and smoking with cognitive decline (16). It is noteworthy that previous studies had some limitations. The majority of the present studies have shown the association of CVH metrics, individually or in various combinations, with cognitive decline (17), but fewer have investigated the effect of CVH metrics, collected completely and measured together as a composite score, on cognitive aging (12). In addition, previous observational studies were implemented by either only using the data of CVH metrics at baseline or without adjusting for time-varying confounders, which may not adequately estimate the influence of CVH on cognitive aging (18, 19). Considering the long duration of cognitive decline (20), conclusions drawn from short follow-up RCTs may also have limited generalizability (19). Nevertheless, RCTs investigating the long-term effects of interventions on CVH metrics are usually impractical because of the high loss to follow-up and high cost (21). Additionally, considering the potential ethno-regional differences (16), there remains a paucity of representative national-based studies that investigate the associations between CVH metrics and cognitive aging among the Chinese older adults. Therefore, it is vital and meaningful to accurately estimate the relationship between interventions on CVH metrics and abnormal cognitive aging using a representative sample based on a longitudinal study.

The parametric g-formula is the appropriate analytic approach when RCTs are not feasible, and standard approaches (e.g., logistic regression) fail to estimate the unbiased effect of intervention in the presence of time-varying confounders if those confounders are affected by prior intervention (19). The g-formula that was first described by Robins allows to simulate different interventions and estimate their effect, regardless of whether the interventions had been implemented in the data used in model construction (22). In particular, the parametric g-formula is fairly effective when evaluating the causal effect of complex joint interventions (23). Recently, the parametric g-formula has been widely used to quantify the long-term effect of hypothetical interventions on outcomes of interest (such as investigating the relationship between sleep disturbance, unemployment, and multimorbidity with depression) in psychiatry, psychology, and epidemiology, which have indicated several types of interventions that can be applied in clinical practice (24, 25). Thus, the parametric g-formula may be a reliable approach for exploring the long-term effect of CVH metrics on abnormal cognitive aging.

Therefore, in the present study, based on a nationally representative 12-year follow-up cohort of older adults in China, we aimed to estimate the effect of CVH metrics on abnormal cognitive aging by using the parametric g-formula.

## Methods

### Data and participants

**Figure 1:**
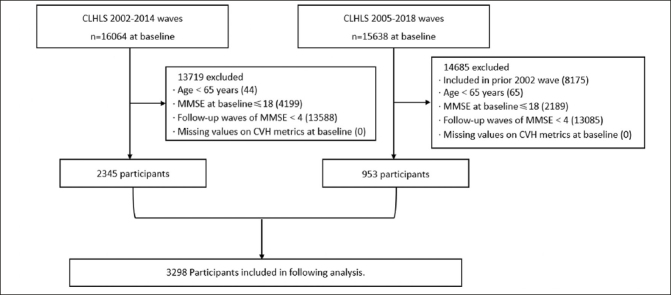
Flow chart of study population from CLHLS Notes: CLHLS, Chinese Longitudinal Healthy Longevity Survey

The present study was based on the Chinese Longitudinal Health Longevity Survey (CLHLS) from wave 3 (2002) to wave 8 (2018). As a nationally representative survey, the CLHLS randomly selected half of the counties and cities in 23 of the 31 provinces, covering approximately 85% of the total population of China. The initial survey was conducted in 1998, and follow-up was performed every two or three years. By 2023, a total of 8 waves (1998, 2000, 2002, 2005, 2008, 2011–2012, 2014, and 2017–2018) were generated. In each wave, new participants were continually enrolled to replace participants lost to attrition, as death and loss to follow-up were inevitable. Throughout each survey, face-to-face interviews were conducted by trained interviewers. In the case of any situation where the participants could not answer the questions independently, a proxy respondent (usually a caregiver or a close relative) was interviewed. However, the questions related to the Mini-Mental State Examination (MMSE) had to be answered by the participants themselves. The present investigation focused on CVH metrics that were completely collected since wave 3. Thus, data from the first two waves were not included in this study. Subjects from the 2002–2014 cohort, supplemented by 953 participants from 2005–2018 cohort, that met the following criteria were eligible for this study: (1) aged 65 years and above, (2) completed at least one CVH metric at baseline, (3) had an MMSE score of 19 or higher at baseline, (4) had no reported dementia at baseline, and (5) completed the MMSE follow-up in at least 4 out of 5 waves. The flow chart of the sample selection process was shown in Figure 1.

The CLHLS was approved by the Biomedical Ethics Committee of Peking University (IRB00001052-13074). Each participant or his/her legal representative provided written informed consent.

### Measures

#### Outcome variable

The Chinese revised version of the MMSE was used to measure cognitive performance at each wave, which has been validated among Chinese older adults (26). The total score ranged from 0 to 30, with a higher score indicating better cognitive performance. As recommended by a previous study (27), a cutoff score > 18 on the MMSE was used to classify participants with normal cognitive performance at baseline. The trajectory groups of cognitive aging were estimated by group-based trajectory model, and the participants were considered to have abnormal cognitive aging if their MMSE scores did not maintain a high level over the follow-up.

#### Intervention variables

According to the AHA’s recommendation (7), CVH was assessed with six metrics, namely, hypertension, diabetes, exercise, body mass index, diet, and smoking. Since total cholesterol was not recorded in each survey, we had to exclude it from the CVH metrics in the present study.

The criteria for each metric were categorized into 2 levels (i.e., ideal or poor): (1) Hypertension: Participants with a systolic blood pressure (SBP) ≥140 mmHg or diastolic blood pressure (DBP) ≥90 mmHg or self-reported hypertension were defined as poor, while others were defined as ideal (28). (2) Diabetes: Participants with self-reported diabetes were defined as poor, while others were defined as ideal. (3) Exercise: Participants who currently engage in regular exercise (referring to intentional physical activities, including walking, running, qigong and so on) were defined as ideal, while others were defined as poor. (4) BMI: Participants with a BMI between 18.5 and 24 kg/m^2^ were defined as ideal, while others were defined as poor (29). (5) Diet: A modified healthy diet score based on the intake frequency of fruits, vegetables, fish, bean products, and tea was defined according to the AHA’s recommended diet goals (7) and “Dietary Guidelines for Chinese residents 2016” (30). The intake frequency of each food group was measured as “always or almost every day”, “sometimes or occasionally”, or “rarely or never”, which received scores of 2, 1, or 0, respectively. The scores were summed and categorized into 2 categories: ideal (7–10) and poor (0–7) (31). (6) Smoking: Participants who never smoked were defined as ideal, while others were defined as poor.

The present study calculated the 6-point CVH total score by summing the number of ideal CVH metrics, which ranged from 0 to 6. CVH was also stratified into three levels: ideal (4–6), intermediate (3), and poor (0–2) (32).

#### Confounders

According to prior literature (33,34), the following variables were identified as potential confounders. The baseline sociodemographic variables included age, sex (male/female), education status (literacy (having schooling education)/illiteracy (without schooling education)), marital status (married/others (i.e., separated, divorced, widowed, and never married)), and living arrangement (with household members/others). The time-varying confounders included psychological resilience, social support, and social engagement.

According to a previous study (33, 34), psychological resilience was assessed by five items (“1. feel the older, the more useless you are”, “2. look on the bright side of things”, “3. feel fearful or anxious”, “4. feel lonely and isolated”, and “5. make own decisions concerning personal affairs”). The response options were coded as “always” (5), “often” (4), “sometimes” (3), “seldom” (2), or “never” (1). Before summation, the first, third, and fourth items were coded reversely. The total score ranged from 5 to 25, with higher scores indicating greater psychological resilience.

As described in a previous study (35), social support consisted of items related to participants’ perceptions of the availability of emotional, instrumental, and financial support from others. In the CLHLS, the data were collected by questioning the availability of a person who would provide help when the participants were experiencing the following situations: when you had problems; when you needed to share some of your thoughts, when you wanted to talk frequently in daily life; when you were sick; when you needed financial support (i.e., the amount of money received from your son and daughter). The responses were coded as “someone” (1) or “nobody” (0). The total score ranged from 0–6, with higher scores indicating greater social support.

Social engagement was measured with three indicators, namely, the frequency of taking part in outdoor activities, playing cards/mahjong, and organized social activities (36). The responses were coded as “always” (2), “sometimes” (1), or “rarely or never” (0). The total score ranged from 0–6, with a higher score indicating greater social engagement.

### Hypothetical interventions on CVH metrics for abnormal cognitive aging

The parametric g-formula was used to estimate the risk of trajectory groups of abnormal cognitive aging and 6 hypothetical single interventions were assessed first. In our models, we made all participants hypothetically (1) not suffer from hypertension, (2) not suffer from diabetes, (3) become current exercising, (4) maintain their BMI between 18.5 and 24 kg/m^2^, (5) have an ideal diet, and (6) never smoke. These interventions were demonstrated at each survey during follow-up. Then, joint interventions consisting of combinations of single interventions that were found to be significant aforementioned were simulated.

### Statistical analysis

The trajectory groups of cognitive aging were identified by group-based trajectory model, which is a type of potential class growth model used to identify the heterogeneity of longitudinal changes and further clusters individuals who follow similar trajectories on an outcome over time. The grouping was achieved by estimating the posterior probabilities of each participant belonging to each potential trajectory group, and those with the highest probability was determined as the final group membership. In the present study, the time metric (0–12) was the number of years since baseline, and the MMSE scores of five waves were used to estimate the trajectory groups of cognitive aging. A censored normal model form was used, considering the continuous outcome of the MMSE score. To find the optimal trajectory groups, the number of groups (2–5 groups) was determined by selecting the smallest absolute Bayesian information criterion (BIC) value. In addition, the average posterior probability (AvePP; above 0.7 indicating good fit) and percentage of group assignments (no less than 5%) were also considered. After determining the number, the shape of each trajectory group was estimated by specifying the order of the polynomial (linear, quadratic, or cubic) (37).

The distribution of baseline characteristics across the trajectory groups of cognitive aging was presented. The chi-square test for categorical variables and the F test for continuous variables were performed to estimate the differences among participants in different trajectory groups. Then, multinormal logistic regression analysis was applied to examine the impact of CVH at baseline on trajectory groups of abnormal cognitive aging. Three models were constructed: unadjusted Model 1; age, sex, education status, and living arrangement were adjusted for in Model 2; and psychological resilience, social support, and social engagement were additionally adjusted for in Model 3. The analyses of the individual CVH metrics in the three models were all adjusted for the rest CVH metrics. Odds ratios (OR) and 95% confidence intervals (95% CI) were reported.

The risk of trajectory groups of abnormal cognitive aging under each hypothetical intervention was estimated by the parametric g-formula. A simplified description of the analytical steps was as follows: (1) fit parametric models for trajectory groups of abnormal cognitive aging, for time-varying covariates (i.e., CVH metrics, psychological resilience, social support, and social engagement); (2) use the observed values of covariates at baseline; (3) use parametric models to estimate the joint distribution of covariates at the next wave; (4) intervene by setting the values of the time-varying exposures (i.e., single CVH metrics or combined CVH metrics) to the values determined by the aforementioned hypothetical interventions (in Section 2.3); and (5) use these new values to simulate the predicted risks of trajectory groups of abnormal cognitive aging by Monte Carlo simulation. To estimate the risk of each trajectory group of abnormal cognitive aging under each hypothetical intervention, steps 1–5 were repeated. The risk ratios (RR) were calculated by comparing the simulated risks under various interventions with the natural course (i.e., simulated risks under no intervention). In addition, 95% CI were computed by nonparametric bootstrapping with 500 samples. Sensitivity analyses were also performed by changing the ordering of time-varying covariates in the model.

The Stata (Stata Corp) Traj plug-in (38) and gformula (39) were used to model the trajectory groups of cognitive aging and g-computation formula. And the trajectory groups plot was also generated by Stata. The descriptive and logistic regression analyses were conducted with SPSS 26.0 (IBM Corp). A two-tailed p value <0.05 was considered to indicate statistical significance.

## Results

### Description of the cohort characteristics

The current study included 3298 participants with a mean (SD) age of 73.9 (8.0) years. At baseline, approximately half of the participants were male (48.3%), had literacy (49.4%), and were married (57.1%). The majority were living with household members (86.7%). For cardiovascular health, the proportions of individuals with ideal, intermediate, and poor level of CVH were 45.4%, 33.2%, and 21.4%, respectively. 3.0% of the participants met all six criteria for ideal CVH metrics (see Table S1 in the Supplementary material). Specifically, 49.6% of the participants had hypertension, 2.1% had diabetes, 37.4% reported currently exercising, 52.1% had an ideal BMI, 43.2% had an ideal dietary pattern, and 61.5% had never smoked (see Table 1).

**Table 1:** General characteristics of participants at baseline

**Variables**	**Total (3298)**	**Trajectory groups**					
		**Stable-high (N=2590)**	**Unstable (N=153)**	**Slow decline (N=337)**	**Rapid decline (N=218)**	***χ***^**2/F**^	**P**
Age (Mean, SD)	73.9 (8.0)	72.1 (6.7)	81.1 (9.4)	79.2 (8.1)	82.3 (8.4)	207.32 ^a^	<0.001
Sex (n, %)							
Male	1593 (48.3)	1353 (52.2)	41 (26.8)	124 (36.8)	75 (34.4)	79.15	<0.001
Female	1705 (51.7)	1237 (47.8)	112 (73.2)	213 (63.2)	143 (65.6)		
Education status (n, %)							
Literacy	1627 (49.4)	1434 (55.5)	36 (23.5)	93 (27.7)	64 (29.6)	176.06	<0.001
Illiteracy	1664 (50.6)	1152 (44.5)	117 (76.5)	243 (72.3)	152 (70.4)		
Marital status (n, %)							
Married	1884 (57.1)	1633 (63.1)	53 (34.6)	135 (40.1)	63 (28.9)	179.70	<0.001
Others	1414 (42.9)	957 (36.9)	100 (65.4)	202 (59.9)	155 (71.1)		
Living arrangement (n, %)							
With household members	2860 (86.7)	2280 (88.0)	122 (79.7)	287 (85.2)	171 (78.4)	24.03	<0.001
Others	438 (13.2)	310 (12.0)	31 (20.3)	50 (14.8)	47 (21.6)		
Psychological resilience (Mean, SD)	19.2 (3.1)	19.5 (3.0)	17.9 (3.0)	18.3 (3.2)	18.0 (3.5)	29.73	<0.001
Social support (Mean, SD)	5.5 (0.9)	5.5 (0.8)	5.4 (1.0)	5.5 (0.9)	5.3 (1.0)	1.63	0.180
Social engagement (Mean, SD)	2.1 (1.2)	2.1 (1.2)	1.8 (1.2)	1.8 (1.1)	1.9 (1.1)	8.50	<0.001
CVH (n, %)							
Ideal	1495 (45.4)	1210 (46.8)	60 (39.2)	130 (38.8)	95 (43.8)	14.96	0.021
Intermediate	1091 (33.2)	852 (32.9)	53 (34.6)	113 (33.7)	72 (33.6)		
Poor	705 (21.4)	524 (20.3)	40 (26.1)	92 (27.5)	49 (22.6)		
Hypertension (n, %)							
Ideal	1635 (50.4)	1311 (50.6)	75 (49.0)	149 (44.2)	100 (45.9)	6.22	0.102
Poor	1663 (49.6)	1279 (49.4)	78 (51.0)	188 (55.8)	118 (54.1)		
Diabetes (n, %)							
Ideal	3228 (97.9)	2533 (97.8)	150 (98.0)	329 (97.6)	216 (99.1)	1.53 ^b^	0.679
Poor	70 (2.1)	57 (2.2)	3 (2.0)	8 (2.4)	2 (0.9)		
Exercise (n, %)							
Ideal	1231 (37.4)	1000 (38.6)	50 (32.7)	100 (29.8)	81 (37.2)	11.5	0.009
Poor	2064 (62.6)	1588 (61.4)	103 (67.3)	236 (70.2)	137 (62.8)		
BMI (n, %)							
Ideal	1717 (52.1)	1388 (53.6)	67 (43.8)	162 (48.1)	100 (46.1)	11.88	0.008
Poor	1580 (47.9)	1202 (46.4)	86 (56.2)	175 (51.9)	117 (53.9)		
Diet (n, %)							
Ideal	1424 (43.2)	1189 (45.9)	50 (32.7)	115 (34.2)	70 (32.1)	36.79	<0.001
Poor	1871 (56.7)	1399 (54.1)	103 (67.3)	221 (65.8)	148 (67.9)		
Smoke (n, %)							
Ideal	2027 (61.5)	1534 (59.3)	104 (68.0)	231 (68.8)	158 (72.5)	26.79	<0.001
Poor	1269 (38.5)	1055 (40.7)	49 (32.0)	105 (31.3)	60 (27.5)		

Notes: CVH, cardiovascular health metric; BMI, body mass index. a.The ages of trajectory groups show unequal variance. Welch’s ANOVA was performed. b. The cells of trajectory classes in diabetes have expected count less than 5. Fisher’s exact test was performed. Result with p-value<0.05 are marked with bold digits.

### Trajectory groups of cognitive aging

**Figure 2:**
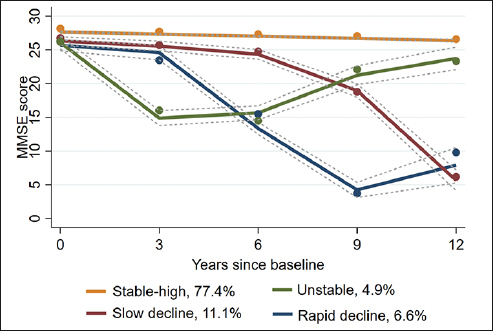
Trajectory groups of cognitive aging over 12 years follow-up Notes: The solid lines show the estimated values, and the dotted lines show the 95% CIs.

Among the 2–5 group, the 4-group showed the best model fit (see Table S2 in the Supplementary material). Thus, four trajectory groups of cognitive aging were identified throughout the 12-year follow-up (see Figure 2): (1) Group 1 was characterized by the maintenance of high MMSE scores over the 12-year follow-up, which was therefore named as Stable-high (77.4%); (2) Group 2 was characterized by a rapid decline in the first two waves and then relative improvement, which was therefore named as Unstable (4.9%); (3) Group 3 was characterized by a steady decline throughout, which was named as Slow decline (11.1%); and (4) Group 4 was characterized by a rapid decline in the first four waves and subsequent maintenance at a low level, which was named as Rapid decline (6.6%). And Unstable, Slow decline, and Rapid decline were considered as trajectory groups of abnormal cognitive aging, while Stable-high as trajectory group of normal cognitive aging.

In addition, the results of univariate analysis across trajectory groups of cognitive aging are shown in Table 1. There were significant differences between the subgroups in terms of age, sex, education status, marital status, living arrangement, psychological resilience, and social engagement. For cardiovascular health, older adults with ideal level of CVH, exercise, BMI, and diet were more likely to be classified as Stable-high trajectory group of cognitive aging.

### Associations between CVH metrics and abnormal cognitive aging

According to the final multinormal logistic regression analysis adjusted for all covariates, when considering the components of CVH, those with poor level of diet (OR=1.75, 95% CI [1.02–2.99]) and smoking (OR=1.98, 95% CI [1.09–3.57]) both showed increased risk for Unstable trajectory group compared with those with ideal level of the corresponding metrics (see Table 2). In addition, when CVH was treated as a continuous variable, older adults with higher CVH scores had a significantly lower risk for Unstable (OR=0.80, 95% CI [0.63–0.99]) and Slow decline (OR=0.88, 95% CI [0.77–0.99]) trajectory groups. Similarly, when CVH was used as a categorical variable, the ideal level of CVH was associated with reduced risks for both Unstable (OR=0.53, 95% CI [0.29–0.99]) and Slow decline (OR=0.70, 95% CI [0.49–1.00]) trajectory groups.

**Table 2:** The association of CVH metrics and the trajectory groups of cognitive aging by multinormal logistic regression

**Characteristic (Ref)**^**a**^	Model 1*			Model 2*			Model 3*		
	**Unstable** ^**b**^ **OR (95% CI)**	**Slow decline** ^**b**^ **OR (95% CI)**	**Rapid decline** ^**b**^ **OR (95% CI)**	**Unstable** ^**b**^ **OR (95% CI)**	**Slow decline** ^**b**^ **OR (95% CI)**	**Rapid decline** ^**b**^ **OR (95% CI)**	**Unstable** ^**b**^ **OR (95% CI)**	**Slow decline** ^**b**^ **OR (95% CI)**	**Rapid decline** ^**b**^ **OR (95% CI)**
Components (Ref) ^a^									
Hypertension (ideal)	1.05 (0.75–1.45)	1.29 (1.03–1.63)	1.21 (0.91–1.60)	0.61 (0.35–1.06)	1.19 (0.93–1.52)	1.12 (0.83–1.51)	0.75 (0.47–1.20)	1.21 (0.92–1.59)	1.00 (0.68–1.47)
Diabetes (ideal)	0.91 (0.28–2.96)	1.14 (0.53–2.43)	0.39 (0.09–1.63)	1.54 (0.45–5.20)	1.72 (0.77–3.84)	0.73 (0.17–3.12)	2.33 (0.52–10.46)	1.02 (0.35–3.01)	1.13 (0.25–5.10)
BMI (18.5–23.9)	1.41 (1.01–1.96)	1.15 (0.91–1.44)	1.28 (0.96–1.69)	1.24 (0.87–1.75)	1.06 (0.83–1.36)	1.17 (0.86–1.59)	1.20 (0.75–1.93)	1.05 (0.80–1.39)	0.93 (0.63–1.38)
Diet (ideal)	1.63 (1.14–2.32)	1.48 (1.16–1.90)	1.77 (1.31–2.40)	1.40 (0.96–2.04)	1.32 (1.02–1.72)	1.56 (1.12–2.18)	1.75 (1.02–2.99)	1.26 (0.94–1.69)	1.49 (0.97–2.29)
Smoke (ideal)	0.74 (0.52–1.05)	0.71 (0.56–0.91)	0.60 (0.44–0.81)	1.53 (0.99–2.35)	1.06 (0.78–1.45)	0.93 (0.64–1.37)	1.98 (1.09–3.57)	1.05 (0.74–1.48)	0.84 (0.51–1.40)
Exercise (ideal)	1.13 (0.79–1.62)	1.34 (1.04–1.73)	0.87 (0.65–1.17)	1.06 (0.72–1.56)	1.23 (0.94–1.63)	0.89 (0.64–1.23)	1.24 (0.71–2.18)	1.13 (0.82–1.55)	1.23 (0.78–1.94)
Composite Score									
CVH (No.)	0.84 (0.73–0.98)	0.83 (0.75–0.92)	0.90 (0.79–1.02)	0.84 (0.71–0.98)	0.84 (0.75–0.94)	0.89 (0.77–1.02)	0.80 (0.63–0.99)	0.88 (0.77–0.99)	0.91 (0.76–1.10)
CVH (category, poor)									
Intermediate	0.82 (0.53–1.25)	0.76 (0.56–1.02)	0.92 (0.63–1.34)	0.73 (0.46–1.14)	0.73 (0.53–1.00)	0.82 (0.54–1.23)	0.85 (0.48–1.52)	0.84 (0.59–1.20)	0.91 (0.53–1.56)
Ideal	0.65 (0.43–0.98)	0.61 (0.46–0.82)	0.84 (0.59–1.20)	0.62 (0.40–0.97)	0.62 (0.46–0.85)	0.78 (0.53–1.16)	0.53 (0.29–0.99)	0.70 (0.49–1.00)	0.97 (0.57–1.63)

Note: CVH, cardiovascular health metrics; BMI, body mass index. *Model1: no covariates adjusted; Model2: adjusted for age, sex, education status, marital status, and living arrangement; Model3: additionally adjusted for psychological resilience, social support, and social engagement. The analysis of the individual CVH metric in three models were all adjusted for the rest CVH metrics. a. (Ref): (Reference). b. reference: Stable-high trajectory group. Result with p-value<0.05 are marked with bold digits.

### The effect of CVH metrics simulated by the parametric g-formula

The simulated risks under no interventions (Unstable: 5.61%; Slow decline: 10.42%; Rapid decline: 7.59%) of the three trajectory groups of abnormal cognitive aging were similar to the observed risks (Unstable: 5.58%; Slow decline: 11.51%; Rapid decline: 7.76%) (see Table 3), which indicated that the g-formula model was satisfactory.

**Table 3:** Risks for trajectory groups of abnormal cognitive aging under hypothetical single and joint interventions

**Intervention**	**Unstable**		**Slow decline**		**Rapid decline**	
	**Risk (95% CI)**^**a**^	**RR (95% CI)**^**b**^	**Risk (95% CI)**^**a**^	**RR (95% CI)**^**b**^	**Risk (95% CI)**^**a**^	**RR (95% CI)**^**b**^
Natural course^c^	5.61 (4.48–6.74)	1.00	10.42 (8.74–12.10)	1.00	7.59 (6.24–8.93)	1.00
Single interventions						
Hypertension	6.19 (4.33–8.07)	1.10 (0.89–1.36)	11.76 (8.43–13.09)	1.13 (0.98–1.31)	5.95 (4.30–7.60)	0.78 (0.64–0.95)
Diabetes	5.10 (3.93–6.27)	0.91 (0.73–1.14)	9.87 (8.23–11.51)	0.95 (0.81–1.10)	6.88 (5.63–8.12)	0.91 (0.75–1.09)
Exercise	3.39 (2.01–4.77)	0.60 (0.47–0.78)	8.44 (5.93–10.95)	0.81 (0.69–0.95)	5.66 (3.88–7.45)	0.75 (0.61–0.91)
BMI	3.40 (2.00–4.77)	0.61 (0.47–0.78)	9.98 (7.85–12.10)	0.96 (0.82–1.12)	5.88 (4.35–7.40)	0.77 (0.64–0.94)
Diet	3.10 (1.44–4.75)	0.55 (0.43–0.71)	6.66 (4.63–8.70)	0.64 (0.54–0.76)	4.13 (2.57–5.69)	0.52 (0.41–0.67)
Smoke	6.49 (5.03–7.95)	1.16 (0.94–1.43)	12.64 (10.74–14.54)	1.21 (1.05–1.40)	8.26 (6.69–9.84)	1.09 (0.91–1.30)
Joint interventions						
BMI + Exercise	2.73 (1.38–4.04)	0.49 (0.37–0.63)	7.17 (4.05–8.18)	0.69 (0.58–0.81)	4.34 (2.56–6.13)	0.57 (0.46–0.71)
Diet + BMI	2.73 (1.34–4.13)	0.49 (0.37–0.63)	6.12 (4.05–8.18)	0.59 (0.49–0.70)	3.56 (1.91–5.20)	0.47 (0.37–0.59)
Diet + Exercise	1.93 (0.61–3.25)	0.34 (0.26–0.46)	4.68 (2.67–6.69)	0.45 (0.37–0.54)	3.31 (1.74–4.88)	0.44 (0.35–0.55)
Diet + BMI + Exercise	1.71 (0.61–2.82)	0.30 (0.23–0.41)	4.65 (2.61–6.68)	0.45 (0.37–0.54)	2.85 (1.43–4.26)	0.38 (0.30–0.48)

Note: RR, risk ratio; BMI, body mass index. a. The observed risks for “Unstable”, “Slow decline”, “Rapid decline” trajectory groups are 5.58%, 11.51%, 7.76%. b. Estimated using the parametric g-formula with fixed covariates: age, sex, education status, marriage status, living arrangement; and time-varying covariates: hypertension, diabetes, exercise, BMI, diet, smoke, psychological resilience, social support, and social engagement. c. As simulated under no intervention.

As illustrated in Table 3, both ideal exercise and diet were effective single interventions on decreasing risks for Unstable (exercise: RR=0.60, 95% CI [0.47–0.78]; diet: RR=0.55, 95% CI [0.43–0.71]), Slow decline (exercise: RR=0.81, 95% CI [0.69–0.95]; diet: RR=0.64, 95% CI [0.54–0.76]), and Rapid decline (exercise: RR=0.75, 95% CI [0.61–0.91]; diet: RR=0.52, 95% CI [0.41–0.67]) trajectory groups. In addition, the single intervention of improving BMI to an ideal level also lowered the risks for Unstable (RR=0.61, 95% CI [0.47–0.78]) and Rapid decline (RR=0.77, 95% CI [0.64–0.94]) trajectory groups. The effects of other CVH metrics were as follows: never smoking for Slow decline trajectory group (RR=1.21, 95% CI [1.05–1.40]), and not suffering from hypertension for Rapid decline trajectory group (RR=0.78, 95% CI [0.64–0.95]). However, hypothetical intervention on diabetes did not substantially reduce the risks for the three trajectory groups of abnormal cognitive aging.

Based on the results of single hypothetical interventions, exercise, BMI, and diet were combined as joint interventions. All the combinations can significantly reduce the risks for Unstable, Slow decline, and Rapid decline trajectory groups (see Table 3). In particular, the “Diet + BMI + Exercise” joint interventions reduced the risk most for Unstable (RR=0.30, 95% CI [0.23–0.41]), Slow decline (RR=0.45, 95% CI [0.37–0.54]), and Rapid decline (RR=0.38, 95% CI [0.30–0.48]) trajectory groups, followed by the combined interventions on “Diet + Exercise”, “Diet + BMI”, and “BMI + Exercise”.

The sensitivity analyses, which examined whether the order of the time-varying covariates could affect the results, showed that the estimates of RR did not change materially (see Table S3–7 in the Supplementary material).

## Discussion

The present investigation, based on the nationally representative 12-year follow-up cohort of the CLHLS, examined the effect of CVH metrics on abnormal cognitive aging. Our findings indicated that single interventions on hypertension, exercise, BMI, and diet significantly reduced the risks for Unstable, Slow decline or Rapid decline trajectory groups of abnormal cognitive aging. Moreover, the joint interventions of exercise, BMI, and diet had stronger effects on decreasing the risk for all trajectory groups of abnormal cognitive aging. To the best of our knowledge, this is the first study to explore hypothetical interventions on CVH metrics for abnormal cognitive aging utilizing the parametric g-formula.

Our study revealed the presence of four distinct trajectory groups of cognitive aging, namely, Stable-high, Unstable, Slow decline, and Rapid decline. And the Unstable, Slow decline, and Rapid decline trajectory groups of cognitive aging accounted for 22.6% of the participants in total, indicating that a considerable proportion of the participants experienced abnormal cognitive aging during their aging process. In previous studies, Slow decline and Rapid decline trajectory groups have been widely reported (5, 6). However, it is noteworthy that an increasing number of studies have shown that a small number of older adults exhibit large fluctuations in addition to an overall pattern of decline in cognitive aging (40, 41). A population-based study conducted in China also revealed that some older adults experienced relative improvement in cognitive function in the context of a downward trend (41). It is noteworthy that a percentage of people diagnosed with mild cognitive impairment returned to normal cognition, which is similar to the pattern of Unstable trajectory group. According to prior research, the APOE-4 carrier status may underlie the transition from MCI back to normal cognition (42). More importantly, the survival analysis of a longitudinal study suggested that participants who met the criteria for MCI and then improved remained at increased risk for retransition to MCI or dementia over the longer term (42). Some previous studies showed that Slow decline and Rapid decline trajectory groups of cognitive aging, as well as Unstable, did exhibit various degrees of cognitive decline (6, 40). This decline was rather uncommon in healthy older adults and most likely reflects pathologic processes in cognitive function (40). Thus, in the present study, it was highly important to consider Unstable, Slow decline, and Rapid decline as trajectory groups of abnormal cognitive aging and to further explore their potential risk factors.

The results from the logistic regression analysis showed that those with poor level of diet and smoking at baseline were more likely to exhibit Unstable trajectory group, which were partly in line with the findings of previous studies (43, 44). Prior study has shown the effect of dietary patterns on the prevention of cognitive decline (14). A meta-analysis concluded that high adherence to the Mediterranean diet, which was widely believed to be a healthy eating pattern, was associated with normal cognitive aging (44). However, a previous study revealed that the protective effect of diet on cognitive decline differed greatly, which might be due to the unique dietary patterns of different regions (21). Thus, in the present study, we defined the diet metric based on the AHA’s recommended diet goals and “Dietary Guidelines for Chinese Residents 2016” to better explore the effect of CVH metrics on abnormal cognitive aging.

Additionally, our study demonstrated that better CVH, measured as a composite continuous or categorical score, was associated with a reduced risk for Unstable trajectory group. We also revealed the significant relationship between CVH and Slow decline trajectory group even when the significant effect of each single CVH metric was not observed, which was consistent with a prospective study based on the Northern Manhattan Study (12). These results indicated that combined CVH metrics may be more effective in reducing the risk of cognitive decline than any single CVH metric. Thus, the association between CVH and cognitive performance is vital for preventing cognitive decline and dementia. Nevertheless, data elucidating the relationship between CVH and abnormal cognitive aging in older adults were still limited. Therefore, our study filled a gap in the understanding of the effect of CVH on abnormal cognitive aging, and had significant implications for the prevention of cognitive decline. It was also found that only 3% of participants in present study met all six criteria for ideal CVH metrics. This finding was in line with the prior researches conducted in Korea and the USA, which reported a low prevalence of meeting all CVH metrics ranged from 0% to 3% (12, 45). Considering the AHA target of improving CVH, it is urgent to make effort to improve the health conscious about CVH among older adults, and explore the practical and feasible interventions on CVH metrics for the prevention of abnormal cognitive aging.

Moreover, the present study further adjusted for time-varying confounders and simulated a 12-year follow-up RCT to estimate the causal effect of each single CVH metric and their combinations on trajectory groups of abnormal cognitive aging. Notably, the results from the parametric g-formula extended the findings of logistic regression analyses, especially when considering the significant effect of CVH metrics on Rapid decline trajectory groups, which was not observed in logistic regression analyses. It confirmed the idea that the parametric g-formula may be a better approach for investigating the long-term causal effect, as analyses using only baseline data could underestimate the effect estimate (46). We independently intervened on all participants to the ideal level of exercise, BMI, and diet, which resulted in significant reductions in the risk of Unstable, Slow decline, and Rapid decline trajectory groups of abnormal cognitive aging. The hypothetical combined intervention of these three factors had a significant effect on decreasing the risk for abnormal cognitive aging, which was consistent with previous evidence (13, 14). A prior RCT showed that older adults who had undergone 2 years of multidomain intervention involving exercise, BMI, diet and other vascular risk monitoring were at lower risk for cognitive decline (14). A prospective study conducted in China also suggested that intervention on exercise was associated with improved cognitive function (13). Previous studies indicated that exercise, obesity, and dietary patterns could all induce changes in the distribution of cerebral blood flow (CBF) and further cause altered functional connectivity (FC) between related brain regions through neurovascular coupling (47, 48). It is widely believed that both altered CBF and aberrant FC are early pathological changes associated with cognitive impairment and dementia (49). Therefore, the CBF and FC theories may underlie the association between these CVH metrics and abnormal cognitive aging.

In addition, the intervention on hypertension significantly reduced the risk in Rapid decline trajectory group but not in Unstable and Slow decline trajectory groups. The relationship between hypertension and cognitive decline is complex. Several RCTs and prospective studies showed that antihypertensive treatments were associated with a reduced risk of dementia (50), while other studies showed only a weak or no association (15, 51). The inconsistencies in the prior literature could be attributed to the fact that the traditional definition of ideal blood pressure may not apply to older adults, as low blood pressure is also associated with increased dementia risk in older individuals (52).

Notably, intervention on keeping the ideal level of smoking was associated with an increased risk for Slow decline trajectory group. The relationship between smoking and cognitive function has not yet been determined. Previous studies showed contradictory results concerning the relationship between smoking and cognitive decline (53, 54). A meta-analysis concluded that the association between smoking and cognitive function may be age dependent. Specifically, smoking, which had a harmful effect on cognitive development in younger children, may have a beneficial effect on cognitive performance in aging adults (53). Jacobsen et al. also suggested that variation in cognitive-related genotypes may underlie cross-study differences in the impact of smoking on cognitive decline (54).

Indeed, the current study did not observe the significant effect of diabetes on abnormal cognitive aging, while several large prospective studies showed that intervention on diabetes was a protective factor for cognitive decline (55, 56). Notably, a meta-analysis revealed that good glycemic control in diabetic patients had a small impact on cognitive function (57). Several previous studies were inclined to focus on the disease duration and glycemic control rather than diabetes itself to investigate the relationship with cognitive decline (58, 59). Thus, the inconsistent results regarding the relationship between diabetes and cognitive decline may be due to the different features of diabetes on which different studies have focused. In addition, although we defined the ideal level of each CVH metric following the recommendations of the AHA, diabetes was self-reported in the present study and likely to be underestimated, which may also contribute to the contradictory results. According to prior research, approximately 10% of Chinese older adults suffered from diabetes in 2000–2004 (60). Therefore, in future studies, more detailed information about diabetes, such as disease duration and glycemic control, should be collected to further explore its relationship with cognitive decline.

The current study had several strengths, including a nationally representative sample, longer follow-up time, standardized survey methods, and a data-driven approach for identifying the trajectory groups of abnormal cognitive aging. We collected CVH metrics, which were measured as individual components and composite scores, to comprehensively investigate their influence on abnormal cognitive aging. Furthermore, we used the parametric g-formula to simulate long-term single and joint interventions on the CVH metrics for trajectory groups of abnormal cognitive aging with adjustment for time-varying confounders. However, limitations still exist. First, the participants included in our research were Chinese older adults with a mean (SD) baseline age of 73.9 (8.0) years. The findings of the present study cannot be generalized to younger adults and non-Chinese population. Second, CVH metrics employed in the present study did not include total cholesterol, which may compromise comparability with future studies using CVH metrics based on the 7 items. The present study also modified the AHA’s criteria for hypertension and diabetes considering that data about individuals’ treatment of these diseases were not collected. And the diabetes metric was solely categorized by self-reporting. These may underestimate the effect of these corresponding interventions for abnormal cognitive aging. Third, cognitive performance was measured solely by the Chinese version of the MMSE. Although MMSE as a relatively belief instrument was widely used to assess the cognitive performance in population-based surveys, it may not be sensitive enough to detect subtle cognitive changes. A more comprehensive neuropsychological assessment may yield more robust results on the relationship between CVH metrics and cognitive aging. Fourth, the validity of our parametric g-formula results was based on the following common assumptions for observational study: no measurement error, no unmeasured confounders, and no model misspecification. Although multiple potential risk factors were adjusted for in the present study to alleviate these problems, measurement error and unmeasured confounders (such as depression and APOE-4 carrier status, which are not available in the present research) were inevitable in observed study. Given the evidence for increased cognitive decline risk from depression and genetic effects, future studies are required to validate the present findings while taking these factors into account. In addition, the absence of model misspecification in the current study has been confirmed, considering that the simulated risks under no interventions (i.e., natural course) from the g-formula were similar to the observed risks. And the sensitivity analyses further showed that the results were robust across different specifications.

## Conclusion

Based on a large population-based longitudinal survey and the application of the parametric g-formula, we identified the trajectory groups of abnormal cognitive aging (i.e., Unstable, Slow decline, and Rapid decline), and further found that hypothetical interventions on CVH metrics such as hypertension, exercise, BMI, and diet were beneficial for protecting older adults from abnormal cognitive aging. Moreover, the joint intervention of exercise, BMI, and diet had a significant effect on risk reduction for all trajectory groups of abnormal cognitive aging. The main findings suggest that efforts to promote interventions on exercise, BMI, and diet, which could be implemented without medicine and are fairly practical and feasible for older adults, may be effective strategies for preventing abnormal cognitive aging and cognitive decline.

## Supplementary material

Supplementary material, approximately 47.9 KB.

## Data Availability

*Data Availability:* The datasets generated and/or analyzed during the current study are available in https://opendata.pku.edu.cn/dataverse/CHADS.

## Notes

*Ethics approval and consent to participate:* The CLHLS was approved by the Biomedical Ethics Committee of Peking University (IRB00001052-13074). Each participant or his/her legal representative provided written informed consent. This study was approved by the Ethics Committee of Peking University Sixth Hospital (2021-Lunshen No. 36).
