# Supplementary material for: Hypothetical Interventions on Cardiovascular Health Metrics for Abnormal Cognitive Aging: An Application of the Parametric g-formula in the CLHLS Cohort Study with 12 Years Follow-Up
Source: J Prev Alzheimers Dis. 2024 Jul 9;11(6):1615–25. doi: 10.14283/jpad.2024.143 (PMC11573857; doi:10.14283/jpad.2024.143)
Supplement: Supplementary file 1 — Supplementary material, approximately 47.9 KB. [file 42414_2024_143_MOESM1_ESM.docx]

**Supplementary material**

| **Table S1.** The distribution of ideal CVH metrics. | | |
| --- | --- | --- |
| CVH (No.) | N | % |
| 0 | 3 | 0.1 |
| 1 | 96 | 2.9 |
| 2 | 606 | 18.4 |
| 3 | 1091 | 33.2 |
| 4 | 910 | 27.7 |
| 5 | 485 | 14.7 |
| 6 | 100 | 3.0 |
| Notes: CVH, cardiovascular health metrics. | | |

| **Table S2.** Average posterior Probabilities and percentages of group assignment, Bayesian information criterion (BIC) statistics of model fit. | | | | | | | |
| --- | --- | --- | --- | --- | --- | --- | --- |
|  | Group1 | Group2 | Group3 | Group4 | Group5 | BIC | 2ΔBIC |
| 2 groups | 0.94 (16.2) | 0.98 (83.8) |  |  |  | -40714.46 | NA |
| 3 groups | 0.89 (3.6) | 0.88 (16.3) | 0.97 (80.1) |  |  | -40450.54 | 527.84 |
| 4 groups | 0.92 (6.6) | 0.83 (11.1) | 0.87 (4.9) | 0.97 (77.4) |  | -39993.29 | 914.50 |
| 5 groups | 0.92 (4.2) | 0.83 (11.4) | 0.93 (3.0) | 0.87 (5.3) | 0.97 (76.1) | -39684.44 | 617.70 |
| Notes: BIC, Bayesian information criterion; NA, not available; 2ΔBIC, twice the difference between the BIC values. | | | | | | | |

| **Table S3.** Sensitivity analysis for risks of abnormal cognitive aging by changing the order of time-varying covariate under interventions. | | | | | | | | |
| --- | --- | --- | --- | --- | --- | --- | --- | --- |
| Intervention | Unstable | |  | Slow decline | |  | Rapid decline | |
|  | Risk (95% CI)^a^ | RR (95% CI)^b^ |  | Risk (95% CI)^a^ | RR (95% CI)^b^ |  | Risk (95% CI)^a^ | RR (95% CI)^b^ |
| **Natural course^c^** | 5.54 (4.42-6.67) | 1.00 |  | 10.66 (8.96-12.36) | 1.00 |  | 7.37 (6.02-8.72) | 1.00 |
| **Single interventions** |  |  |  |  |  |  |  |  |
| Hypertension | 6.05 (4.19-7.91) | 1.09 (0.88-1.35) |  | 10.01 (7.71-12.30) | 0.94 (0.81-1.09) |  | 6.02 (4.37-7.67) | **0.82 (0.67-0.99)** |
| Diabetes | 5.36 (4.21-6.51) | 0.97 (0.78-1.21) |  | 10.86 (9.25-12.48) | 1.02 (0.88-1.18) |  | 6.62 (5.39-7.86) | 0.90 (0.74-1.09) |
| Exercise | 3.54 (0.22-4.90) | **0.64 (0.50-0.82)** |  | 8.27 (5.75-10.79) | **0.78 (0.66-0.91)** |  | 5.52 (3.76-7.28) | **0.75 (0.61-0.92)** |
| BMI | 3.46 (2.08-4.84) | **0.62 (0.49-0.80)** |  | 9.70 (7.56-11.84) | 0.91 (0.78-1.06) |  | 5.80 (4.28-7.33) | **0.79 (0.65-0.96)** |
| Diet | 2.92 (1.27-4.56) | **0.53 (0.41-0.68)** |  | 6.22 (4.16-8.28) | **0.58 (0.49-0.69)** |  | 4.24 (2.67-5.81) | **0.58 (0.46-0.71)** |
| Smoke | 6.34 (4.89-7.80) | 1.14 (0.93-1.41) |  | 12.33 (10.41-14.26) | **1.16 (1.00-1.33)** |  | 8.69 (7.11-10.27) | 1.18 (0.99-1.41) |
| **Joint interventions** |  |  |  |  |  |  |  |  |
| BMI + Exercise | 2.70 (1.35-4.05) | **0.49 (0.38-0.63)** |  | 7.24 (4.55-9.94) | **0.68 (0.58-0.80)** |  | 4.38 (2.59-6.17) | **0.59 (0.48-0.74)** |
| Diet + BMI | 2.66 (1.28-4.05) | **0.48 (0.37-0.62)** |  | 6.29 (4.24-8.33) | **0.59 (0.50-0.70)** |  | 3.45 (1.80-5.10) | **0.47 (0.37-0.59)** |
| Diet + Exercise | 1.90 (0.58-3.22) | **0.34 (0.26-0.46)** |  | 4.82 (2.85-6.78) | **0.45 (0.38-0.55)** |  | 3.24 (1.67-4.81) | **0.44 (0.35-0.56)** |
| Diet + BMI + Exercise | 1.60 (0.50-2.71) | **0.29 (0.21-0.39)** |  | 4.33 (2.28-6.40) | **0.41 (0.34-0.49)** |  | 3.06 (1.64-4.49) | **0.42 (0.33-0.53)** |
| **Note:** RR, risk ratio; BMI, body mass index.  The order of time-varying covariate in this analysis was diabetes, exercise, BMI, smoke, diet, hypertension, social engagement, social support, psychological resilience.  ^a^ The observed risks of “Unstable”, “Slow decline”, “Rapid decline” trajectory groups are 5.58%, 11.51%, 7.76%.  ^b^ Estimated using the parametric g-formula with fixed covariates: age, sex, education status, marriage status, living arrangement; and time-varying covariates: hypertension, diabetes, exercise, BMI, diet, smoke, psychological resilience, social support, and social engagement.  ^c^ As simulated under no intervention. | | | | | | | | |

| **Table S4.** Sensitivity analysis for risks of abnormal cognitive aging by changing the order of time-varying covariate under interventions. | | | | | | | | |
| --- | --- | --- | --- | --- | --- | --- | --- | --- |
| Intervention | Unstable | |  | Slow decline | |  | Rapid decline | |
|  | Risk (95% CI)^a^ | RR (95% CI)^b^ |  | Risk (95% CI)^a^ | RR (95% CI)^b^ |  | Risk (95% CI)^a^ | RR (95% CI)^b^ |
| **Natural course^c^** | 5.54 (4.39-6.69) | 1.00 |  | 10.38 (8.74-12.03) | 1.00 |  | 7.62 (6.28-8.96) | 1.00 |
| **Single interventions** |  |  |  |  |  |  |  |  |
| Hypertension | 6.81 (5.01-8.63) | 1.23 (1.00-1.51) |  | 10.39 (8.05-12.73) | 1.00 (0.86-1.17) |  | 6.23 (4.58-7.88) | **0.82 (0.67-0.99)** |
| Diabetes | 5.47 (4.23-6.70) | 0.99 (0.79-1.23) |  | 10.66 (8.94-12.38) | 1.03 (0.88-1.20) |  | 7.09 (5.87-8.31) | 0.93 (0.77-1.12) |
| Exercise | 3.39 (1.99-4.79) | **0.61 (0.48-0.79)** |  | 8.40 (5.89-10.92) | **0.81 (0.69-0.95)** |  | 5.24 (3.48-6.99) | **0.69 (0.56-0.84)** |
| BMI | 3.65 (2.27-5.02) | **0.66 (0.52-0.84)** |  | 9.81 (7.70-11.91) | 0.95 (0.81-1.11) |  | 5.66 (4.16-7.16) | **0.74 (0.61-0.91)** |
| Diet | 3.03 (1.43-4.62) | **0.55 (0.42-0.71)** |  | 6.80 (4.75-8.85) | **0.66 (0.55-0.78)** |  | 4.34 (2.74-5.95) | **0.57 (0.46-0.70)** |
| Smoke | 5.03 (3.63-6.43) | 0.91 (0.73-1.14) |  | 12.06 (10.20-13.92) | **1.16 (1.00-1.35)** |  | 8.55 (6.96-10.14) | 1.12 (0.94-1.34) |
| **Joint interventions** |  |  |  |  |  |  |  |  |
| BMI + Exercise | 2.81 (1.45-4.16) | **0.51 (0.39-0.66)** |  | 7.55 (4.84-10.26) | **0.73 (0.61-0.86)** |  | 4.27 (2.46-6.09) | **0.56 (0.45-0.69)** |
| Diet + BMI | 2.84 (1.44-4.25) | **0.51 (0.39-0.67)** |  | 6.25 (4.20-8.31) | **0.60 (0.50-0.72)** |  | 3.49 (1.83-5.15) | **0.46 (0.37-0.57)** |
| Diet + Exercise | 2.04 (0.73-3.35) | **0.37 (0.28-0.49)** |  | 4.65 (2.62-6.68) | **0.45 (0.37-0.54)** |  | 3.21 (1.64-4.77) | **0.42 (0.33-0.53)** |
| Diet + BMI + Exercise | 1.60 (0.51-2.70) | **0.29 (0.21-0.39)** |  | 4.61 (2.60-6.62) | **0.44 (0.37-0.54)** |  | 2.99 (1.53-4.45) | **0.39 (0.31-0.50)** |
| **Note:** RR, risk ratio; BMI, body mass index.  The order of time-varying covariate in this analysis was diet, exercise, BMI, hypertension, smoke, diabetes, social engagement, psychological resilience, social support.  ^a^ The observed risks for “Unstable”, “Slow decline”, “Rapid decline” trajectory groups are 5.58%, 11.51%, 7.76%.  ^b^ Estimated using the parametric g-formula with fixed covariates: age, sex, education status, marriage status, living arrangement; and time-varying covariates: hypertension, diabetes, exercise, BMI, diet, smoke, psychological resilience, social support, and social engagement.  ^c^ As simulated under no intervention. | | | | | | | | |

| **Table S5.** Sensitivity analysis for risks of abnormal cognitive aging by changing the order of time-varying covariate under interventions. | | | | | | | | |
| --- | --- | --- | --- | --- | --- | --- | --- | --- |
| Intervention | Unstable | |  | Slow decline | |  | Rapid decline | |
|  | Risk (95% CI)^a^ | RR (95% CI)^b^ |  | Risk (95% CI)^a^ | RR (95% CI)^b^ |  | Risk (95% CI)^a^ | RR (95% CI)^b^ |
| **Natural course^c^** | 5.36 (4.23-6.48) | 1.00 |  | 10.63 (8.95-12.30) | 1.00 |  | 7.73 (6.39-9.07) | 1.00 |
| **Single interventions** |  |  |  |  |  |  |  |  |
| Hypertension | 6.53 (4.73-8.32) | 1.22 (0.99-1.50) |  | 10.39 (8.01-12.76) | 0.98 (0.84-1.14) |  | 6.34 (4.71-7.97) | **0.82 (0.68-0.99)** |
| Diabetes | 5.61 (4.38-6.85) | 1.05 (0.84-1.30) |  | 10.83 (9.13-12.53) | 1.02 (0.88-1.18) |  | 7.16 (5.91-8.40) | 0.93 (0.77-1.11) |
| Exercise | 3.46 (2.05-4.87) | **0.65 (0.50-0.83)** |  | 8.40 (5.85-10.95) | **0.79 (0.67-0.93)** |  | 5.16 (3.41-6.92) | **0.67 (0.55-0.82)** |
| BMI | 3.57 (2.21-4.94) | **0.67 (0.52-0.85)** |  | 9.91 (7.76-12.05) | 0.93 (0.80-1.08) |  | 5.80 (4.32-7.29) | **0.75 (0.62-0.91)** |
| Diet | 3.17 (1.57-4.78) | **0.59 (0.46-0.76)** |  | 6.18 (4.16-8.21) | **0.58 (0.49-0.69)** |  | 4.06 (2.45-5.67) | **0.53 (0.42-0.65)** |
| Smoke | 5.14 (3.75-6.53) | 0.96 (0.77-1.20) |  | 12.09 (10.26-13.93) | 1.14 (0.99-1.31) |  | 8.44 (6.85-10.03) | 1.09 (0.92-1.30) |
| **Joint interventions** |  |  |  |  |  |  |  |  |
| BMI + Exercise | 2.77 (1.41-4.13) | **0.52 (0.40-0.67)** |  | 7.62 (4.91-10.32) | **0.72 (0.61-0.84)** |  | 4.27 (2.47-6.08) | **0.55 (0.45-0.68)** |
| Diet + BMI | 2.73 (1.33-4.14) | **0.51 (0.39-0.67)** |  | 6.66 (4.60-8.72) | **0.63 (0.53-0.74)** |  | 3.77 (2.13-5.42) | **0.49 (0.39-0.61)** |
| Diet + Exercise | 2.15 (0.83-3.47) | **0.40 (0.30-0.53)** |  | 4.82 (2.80-6.84) | **0.45 (0.38-0.55)** |  | 3.21 (1.64-4.77) | **0.42 (0.33-0.52)** |
| Diet + BMI + Exercise | 1.71 (0.62-2.80) | **0.32 (0.23-0.43)** |  | 4.37 (2.33-6.41) | **0.41 (0.34-0.50)** |  | 2.88 (1.43-4.34) | **0.37 (0.29-0.47)** |
| **Note:** RR, risk ratio; BMI, body mass index.  The order of time-varying covariate in this analysis was diet, exercise, BMI, hypertension, smoke, diabetes, social engagement, social support, psychological resilience.  ^a^ The observed risks for “Unstable”, “Slow decline”, “Rapid decline” trajectory groups are 5.58%, 11.51%, 7.76%.  ^b^ Estimated using the parametric g-formula with fixed covariates: age, sex, education status, marriage status, living arrangement; and time-varying covariates: hypertension, diabetes, exercise, BMI, diet, smoke, psychological resilience, social support, and social engagement.  ^c^ As simulated under no intervention. | | | | | | | | |

| **Table S6.** Sensitivity analysis for risks of abnormal cognitive aging by changing the order of time-varying covariate under interventions. | | | | | | | | |
| --- | --- | --- | --- | --- | --- | --- | --- | --- |
| Intervention | Unstable | |  | Slow decline | |  | Rapid decline | |
|  | Risk (95% CI)^a^ | RR (95% CI)^b^ |  | Risk (95% CI)^a^ | RR (95% CI)^b^ |  | Risk (95% CI)^a^ | RR (95% CI)^b^ |
| **Natural course^c^** | 5.61 (4.49-6.74) | 1.00 |  | 10.86 (9.17-12.56) | 1.00 |  | 7.69 (6.36-9.02) | 1.00 |
| **Single interventions** |  |  |  |  |  |  |  |  |
| Hypertension | 6.56 (4.77-8.35) | 1.17 (0.95-1.44) |  | 9.91 (7.60-12.21) | 0.91 (0.78-1.06) |  | 5.91 (4.28-7.54) | **0.77 (0.63-0.93)** |
| Diabetes | 5.54 (4.40-6.69) | 0.99 (0.79-1.23) |  | 11.10 (9.47-12.74) | 1.02 (0.88-1.18) |  | 6.80 (5.54-8.06) | 0.88 (0.73-1.07) |
| Exercise | 3.06 (1.59-4.53) | **0.55 (0.42-0.70)** |  | 8.03 (5.48-10.58) | **0.74 (0.63-0.87)** |  | 5.80 (4.02-7.59) | **0.75 (0.62-0.92)** |
| BMI | 4.23 (2.83-5.63) | **0.75 (0.60-0.95)** |  | 9.77 (7.71-11.84) | 0.90 (0.77-1.05) |  | 5.73 (4.25-7.22) | **0.75 (0.61-0.91)** |
| Diet | 3.03 (1.39-4.66) | **0.54 (0.42-0.70)** |  | 6.05 (3.99-8.10) | **0.56 (0.47-0.66)** |  | 4.27 (2.74-5.81) | **0.56 (0.45-0.69)** |
| Smoke | 6.09 (4.58-7.59) | 1.09 (0.88-1.34) |  | 12.09 (10.08-14.10) | 1.11 (0.97-1.28) |  | 8.62 (7.07-10.17) | 1.12 (0.94-1.34) |
| **Joint interventions** |  |  |  |  |  |  |  |  |
| BMI + Exercise | 2.84 (1.50-4.19) | **0.51 (0.39-0.66)** |  | 7.00 (4.32-9.69) | **0.64 (0.55-0.76)** |  | 4.49 (2.74-6.24) | **0.58 (0.47-0.72)** |
| Diet + BMI | 2.59 (1.19-3.99) | **0.46 (0.35-0.60)** |  | 6.52 (4.45-8.61) | **0.60 (0.51-0.71)** |  | 3.49 (1.88-5.10) | **0.45 (0.36-0.57)** |
| Diet + Exercise | 1.93 (0.60-3.26) | **0.34 (0.26-0.46)** |  | 4.99 (2.99-6.99) | **0.46 (0.38-0.55)** |  | 3.06 (1.50-4.63) | **0.40 (0.31-0.50)** |
| Diet + BMI + Exercise | 1.68 (0.58-2.77) | **0.30 (0.22-0.41)** |  | 4.51 (2.46-6.56) | **0.42 (0.34-0.50)** |  | 2.81 (1.39-4.23) | **0.37 (0.29-0.46)** |
| **Note:** CVH, cardiovascular health metrics; BMI, body mass index.  The order of time-varying covariate in this analysis was hypertension, diabetes, exercise, BMI, diet, smoke, social engagement, social support, psychological resilience.  ^a^ The observed risks for “Unstable”, “Slow decline”, “Rapid decline” trajectory groups are 5.58%, 11.51%, 7.76%.  ^b^ Estimated using the parametric g-formula with fixed covariates: age, sex, education status, marriage status, living arrangement; and time-varying covariates: hypertension, diabetes, exercise, BMI, diet, smoke, psychological resilience, social support, and social engagement.  ^c^ As simulated under no intervention. | | | | | | | | |

| **Table S7.** Sensitivity analysis for risks of abnormal cognitive aging by changing the order of time-varying covariate under interventions. | | | | | | | | |
| --- | --- | --- | --- | --- | --- | --- | --- | --- |
| Intervention | Unstable | |  | Slow decline | |  | Rapid decline | |
|  | Risk (95% CI)^a^ | RR (95% CI)^b^ |  | Risk (95% CI)^a^ | RR (95% CI)^b^ |  | Risk (95% CI)^a^ | RR (95% CI)^b^ |
| **Natural course^c^** | 5.80 (4.64-6.95) | 1.00 |  | 10.45 (8.80-12.11) | 1.00 |  | 7.41 (6.09-8.72) | 1.00 |
| **Single interventions** |  |  |  |  |  |  |  |  |
| Hypertension | 6.49 (4.68-8.30) | 1.12 (0.91-1.38) |  | 10.35 (8.07-12.64) | 0.99 (0.85-1.15) |  | 6.23 (4.58-7.88) | 0.84 (0.69-1.02) |
| Diabetes | 5.18 (4.04-6.31) | 0.89 (0.72-1.11) |  | 11.14 (9.47-12.80) | 1.07 (0.92-1.24) |  | 7.02 (5.77-8.26) | 0.95 (0.79-1.11) |
| Exercise | 3.10 (1.65-4.54) | **0.53 (0.41-0.69)** |  | 7.99 (5.48-10.51) | **0.76 (0.65-0.90)** |  | 5.27 (3.53-7.01) | **0.71 (0.58-0.87)** |
| BMI | 4.30 (2.92-5.69) | **0.74 (0.59-0.93)** |  | 9.87 (7.81-11.94) | 0.94 (0.81-1.10) |  | 5.34 (3.83-6.86) | **0.72 (0.59-0.88)** |
| Diet | 3.03 (1.39-4.66) | **0.52 (0.40-0.67)** |  | 6.66 (4.61-8.71) | **0.64 (0.54-0.76)** |  | 4.34 (2.72-5.97) | **0.59 (0.47-0.73)** |
| Smoke | 5.87 (4.37-7.37) | 1.01 (0.82-1.25) |  | 11.89 (9.92-13.86) | 1.14 (0.98-1.31) |  | 8.51 (6.94-10.09) | 1.15 (0.96-1.37) |
| **Joint interventions** |  |  |  |  |  |  |  |  |
| BMI + Exercise | 2.81 (1.45-4.16) | **0.48 (0.37-0.63)** |  | 7.21 (4.47-9.94) | **0.69 (0.58-0.81)** |  | 4.49 (2.74-6.24) | **0.61 (0.49-0.75)** |
| Diet + BMI | 2.59 (1.20-3.97) | **0.45 (0.34-0.58)** |  | 6.22 (4.13-8.30) | **0.60 (0.50-0.71)** |  | 3.60 (1.99-5.21) | **0.49 (0.39-0.61)** |
| Diet + Exercise | 2.01 (0.67-3.34) | **0.35 (0.26-0.46)** |  | 4.92 (2.90-6.94) | **0.47 (0.39-0.57)** |  | 3.21 (1.64-4.77) | **0.43 (0.34-0.55)** |
| Diet + BMI + Exercise | 1.68 (0.59-2.76) | **0.29 (0.21-0.39)** |  | 4.82 (2.79-6.94) | **0.46 (0.38-0.56)** |  | 2.71 (1.27-4.15) | **0.37 (0.29-0.47)** |
| **Note:** CVH, cardiovascular health metrics; BMI, body mass index.  The order of time-varying covariate in this analysis was hypertension, diabetes, exercise, BMI, diet, smoke, social engagement, psychological resilience, social support.  ^a^ The observed risks for “Unstable”, “Slow decline”, “Rapid decline” trajectory groups are 5.58%, 11.51%, 7.76%.  ^b^ Estimated using the parametric g-formula with fixed covariates: age, sex, education status, marriage status, living arrangement; and time-varying covariates: hypertension, diabetes, exercise, BMI, diet, smoke, psychological resilience, social support, and social engagement.  ^c^ As simulated under no intervention. | | | | | | | | |
